# Early Thumb Carpometacarpal Subluxation Stabilized with a Mini TightRope: A Report of Two Cases

**DOI:** 10.1016/j.jhsg.2023.10.006

**Published:** 2023-11-24

**Authors:** Christopher R. Gajewski, Neil F. Jones

**Affiliations:** ∗ Department of Orthopedic Surgery, University of California, Los Angeles, CA

**Keywords:** Basilar thumb arthritis, Hypermobility, Instability, Suspensionplasty

## Abstract

Two patients with thumb carpometacarpal instability were stabilized using a suture suspension device. Both patients had symptomatic thumb carpometacarpal instability in the setting of clinical hyperlaxity without known connective tissue disorder that was recalcitrant to nonsurgical modalities. Both patients had significant, lasting improvement in their pain and function with excellent radiographic outcomes. Suture suspension as a treatment for thumb carpometacarpal instability with an intact trapezium is an effective alternative to ligamentous reconstruction that avoids donor site morbidity and may have added benefit in patients with underlying ligamentous laxity.

The thumb carpometacarpal (CMC) joint is unique with extensive mobility, yet little intrinsic stability given the shallow articular surface.^1^ The thumb CMC joint primarily relies on a complex interplay of the capsule, ligaments, muscles, and tendons for stability throughout range of motion and load-bearing activities.^2^ Instability at this joint has many etiologies, including connective tissue disorders, overuse, and trauma.^3^ Correct identification of the etiology is paramount to a successful treatment outcome, which is primarily directed at creating a stable, pain-free digit.

Various treatment options exist for the management of thumb CMC instability, ranging from nonsurgical methods to surgical intervention. Traditionally, surgical stabilization involves rerouting a distally based slip of the flexor carpi radialis tendon through the base of the first metacarpal, as originally described by Eaton and Littler.^4^ Numerous modifications have been proposed in recent decades; however, there are minimal data surrounding efficacy to guide treatment decisions.^5^ Recently, suture-button suspensionplasty has been used extensively in the operative management of basilar thumb arthritis in conjunction with trapieziectomy.^6^

This report presents the novel use of a thumb to index metacarpal suspensionplasty in two patients using the Arthrex Mini TightRope for the management of thumb CMC instability with an intact trapezium.

### Statement of consent

Both patients/guardians provided written consent for the collection of personal health data and submission for publication.

## Case Report

### Case A

A 14-year-old right hand–dominant female student presented with a 15-month history of atraumatic, progressively worsening right thumb pain causing difficulty with pinch and heavy grip. An outside orthopedic hand surgeon diagnosed her with thumb CMC instability seen under fluoroscopy and immobilized her in a thumb spica cast for 1 month, followed by 3 months of physical therapy for thumb CMC stabilization exercises, which failed to alleviate her symptoms.

Examination revealed hyperextension at both elbows and knees with a Beighton hypermobility score of 9/9. The right thumb had evidence of volar and radial subluxation of her CMC joint, but the grind test was negative. Ligamentous testing of the ipsilateral thumb metacarpophalangeal (MCP) joint demonstrated laxity with radial deviation consistent with an ulnar collateral ligament (UCL) injury.

**Figure 1:**
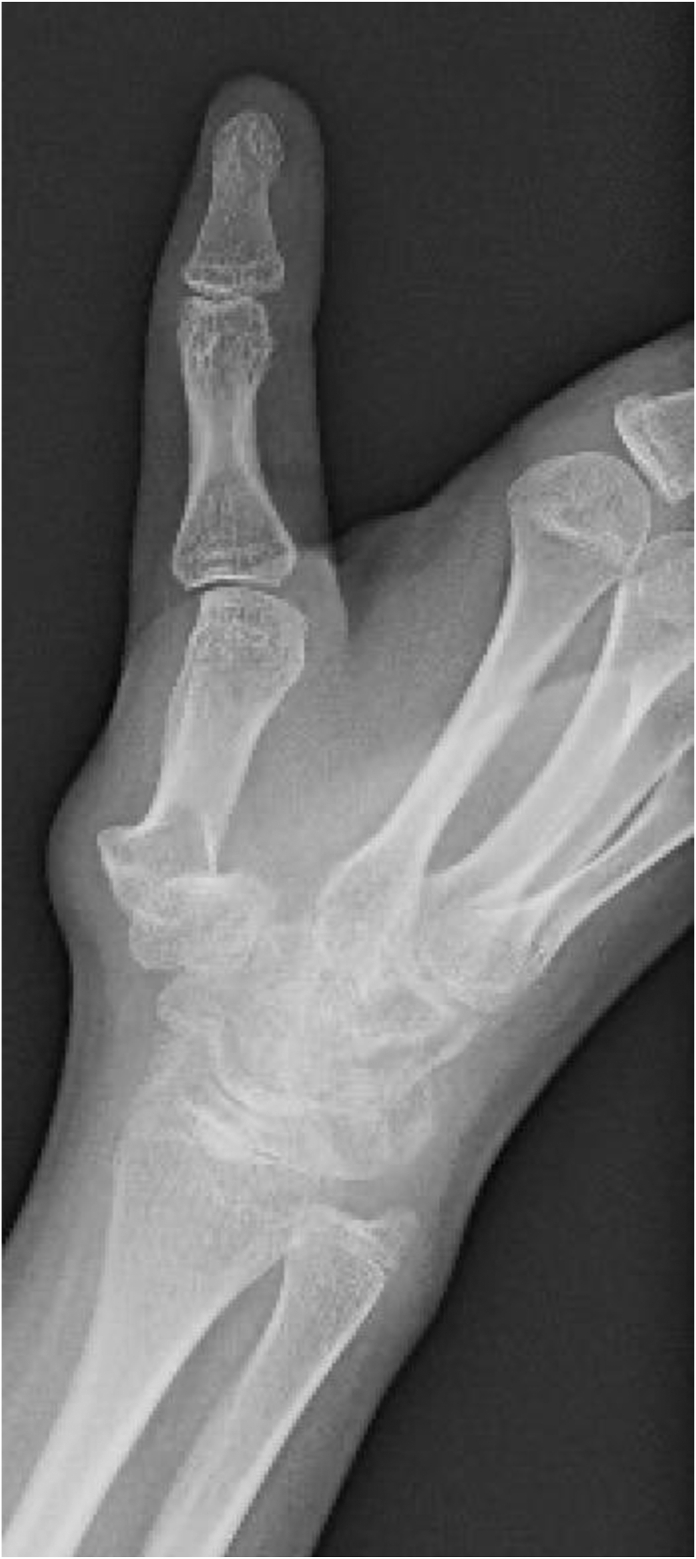
Anteroposterior radiograph of the right thumb demonstrating radial deviation of the thumb CMC and MCP joints.

**Figure 2:**
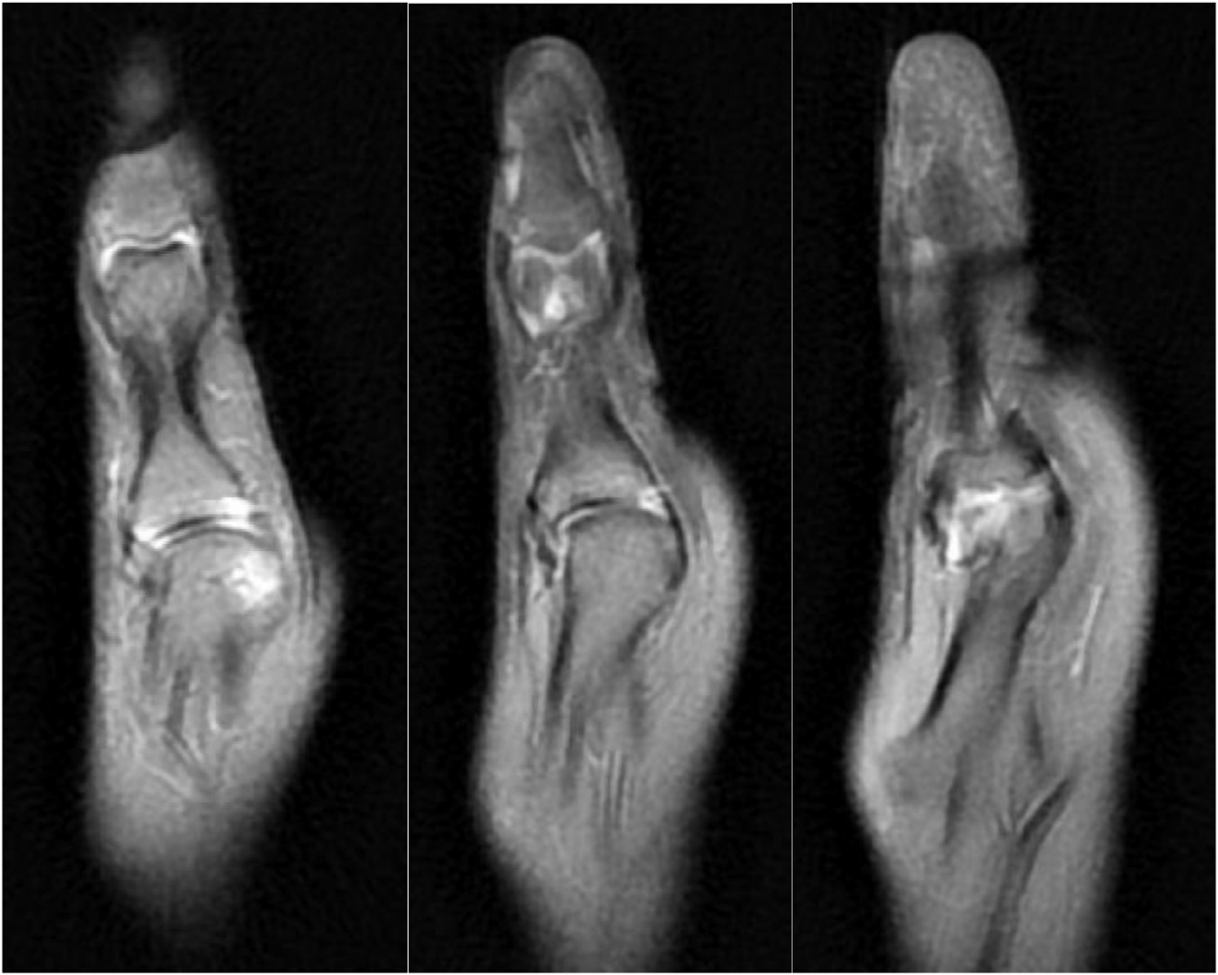
Sequential right thumb coronal sections of a proton density sequence demonstrating disruption of the MCP UCL with hyperintensity of the bony insertion on the metacarpal head.

Plain radiographs demonstrated radial subluxation of the thumb CMC and MCP joints with magnetic resonance imaging evidence of thumb UCL disruption (Figure 1, Figure 2). Initially, the patient was placed in a long opponens splint and referred to pediatric rheumatology for consultation given evidence of hypermobility, but an underlying connective tissue disorder could not be diagnosed.

Given the pathology at both the thumb CMC and MCP joint, operative stabilization was recommended to avoid arthrodesis of one or both joints, which would severely restrict thumb motion and function. Stabilization of the thumb with an Eaton-Littler procedure versus a suspensionplasty was discussed, and the patient elected to proceed with the latter treatment option.

#### Surgical technique

A dorsal longitudinal incision was made in the interval between the extensor pollicis longus and extensor pollicis brevis tendons was used to expose the base of the first metacarpal and trapezium. The first CMC joint was manually reduced and temporarily stabilized with a K-wire. A second longitudinal incision was made over the base of the second metacarpal to expose the ulnar border. The base of the first metacarpal was then secured to the base of the second metacarpal with placement of an Arthrex Mini TightRope device. An additional K-wire was then placed from the first metacarpal into the second metacarpal. A second Mini TightRope device was placed distally for added stability given the patient’s young age and high functional demands. The patient was immobilized in a thumb spica splint.

#### Postoperative course

**Figure 3:**
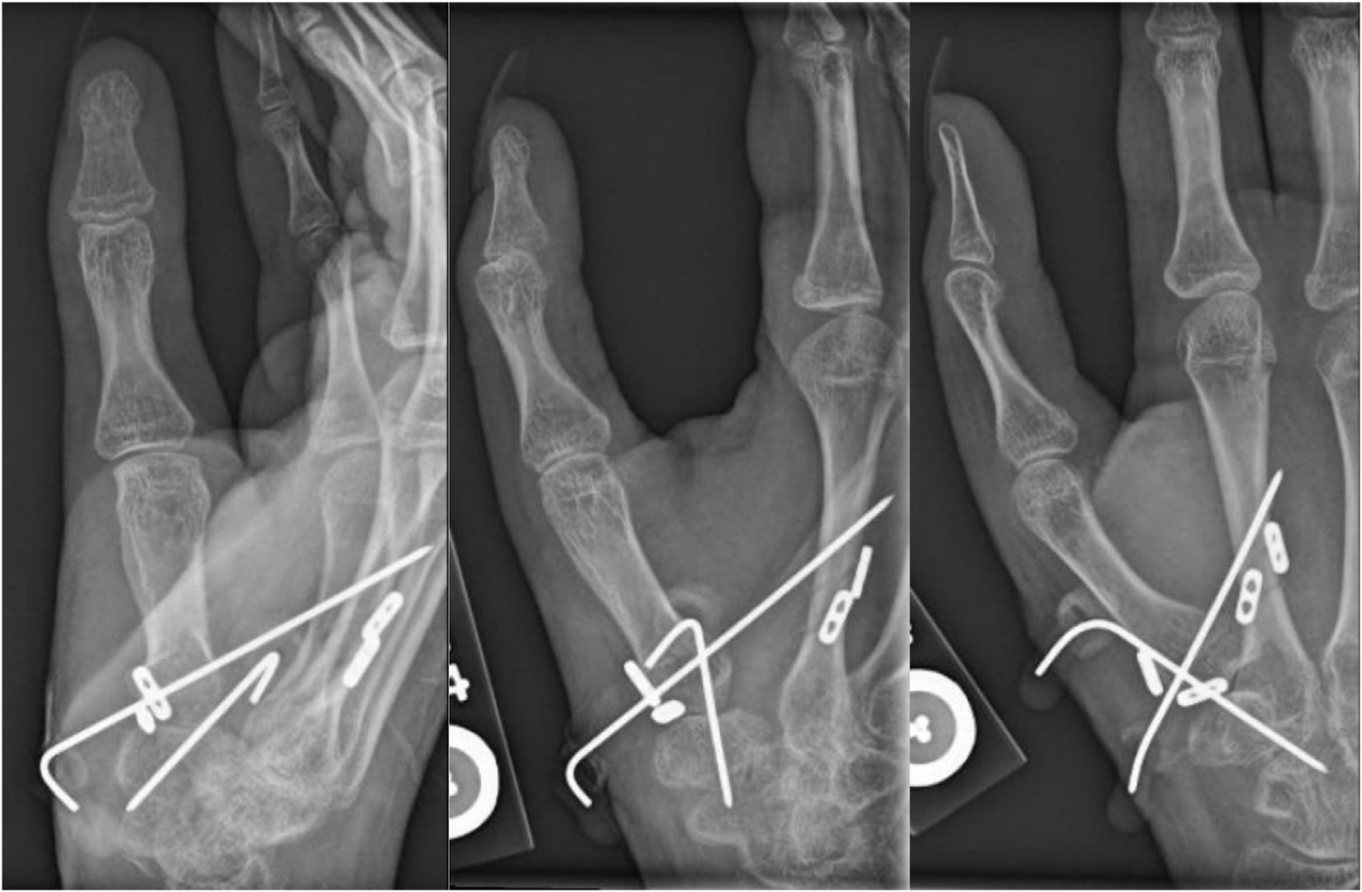
Postoperative day 13 posteroanterior, oblique and lateral right thumb radiographs showing reduction of the thumb CMC joint stabilized with two Arthrex Mini Tightropes and two K-wires.

**Figure 4:**
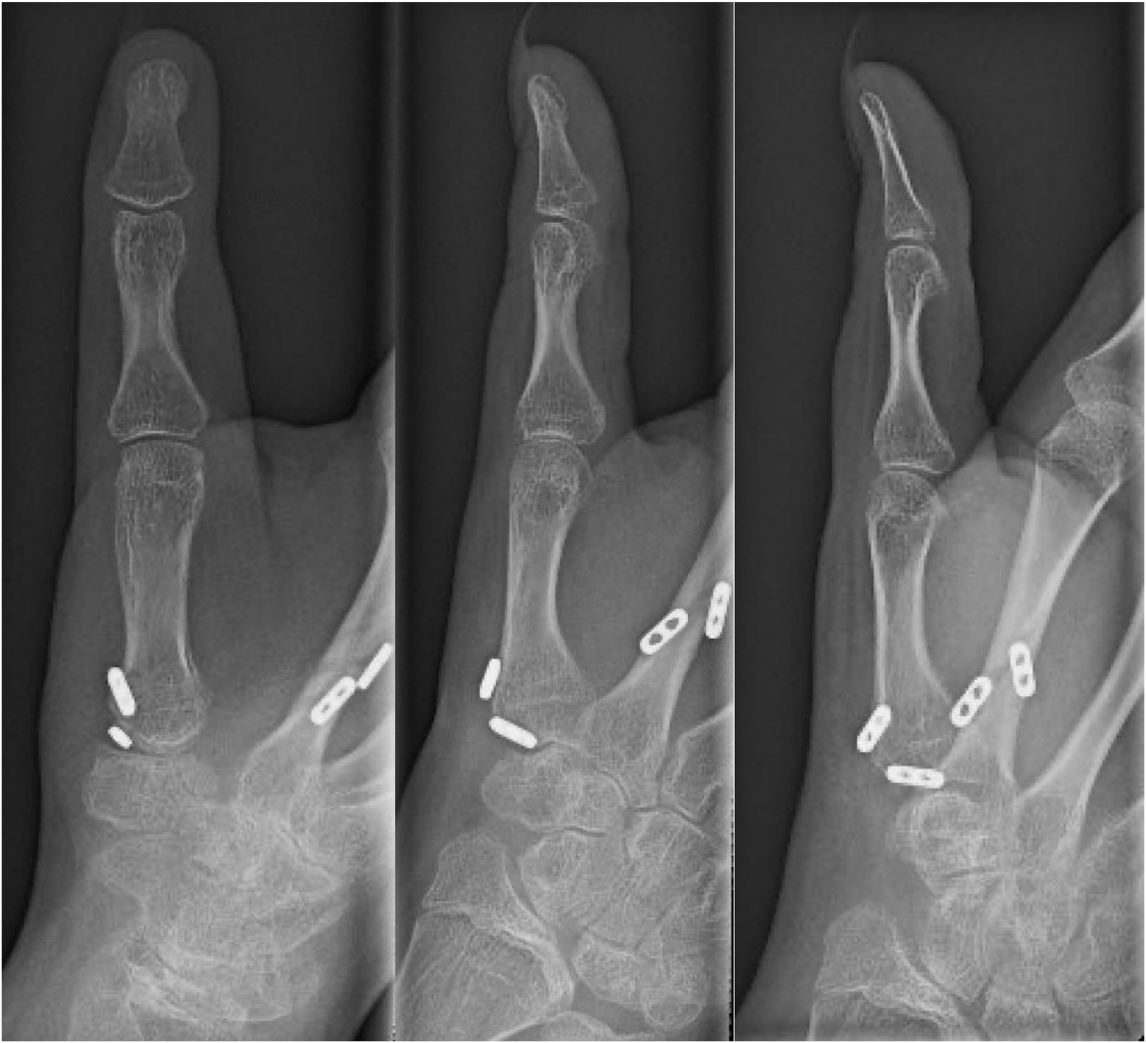
Fourteen-week postoperative posteroanterior, oblique and lateral radiographs of the right thumb demonstrating anatomic alignment of the thumb CMC joint with interval K-wire removal.

On postoperative day 13, radiographs demonstrated stable alignment, and she was transitioned to a short arm thumb spica cast for an additional 8 weeks (Fig. 3). When immobilization was discontinued, the K-wires were removed. The patient then began active range of motion exercises of her thumb, and by 14 weeks, the patient had no pain and was using her right hand for light activities. The thumb was in satisfactory alignment, and the patient was able to oppose her thumb to the base of the small finger (Kapandji score 10). Radiographs demonstrated stable hardware with anatomic alignment of the thumb CMC joint (Fig. 4).

**Figure 5:**
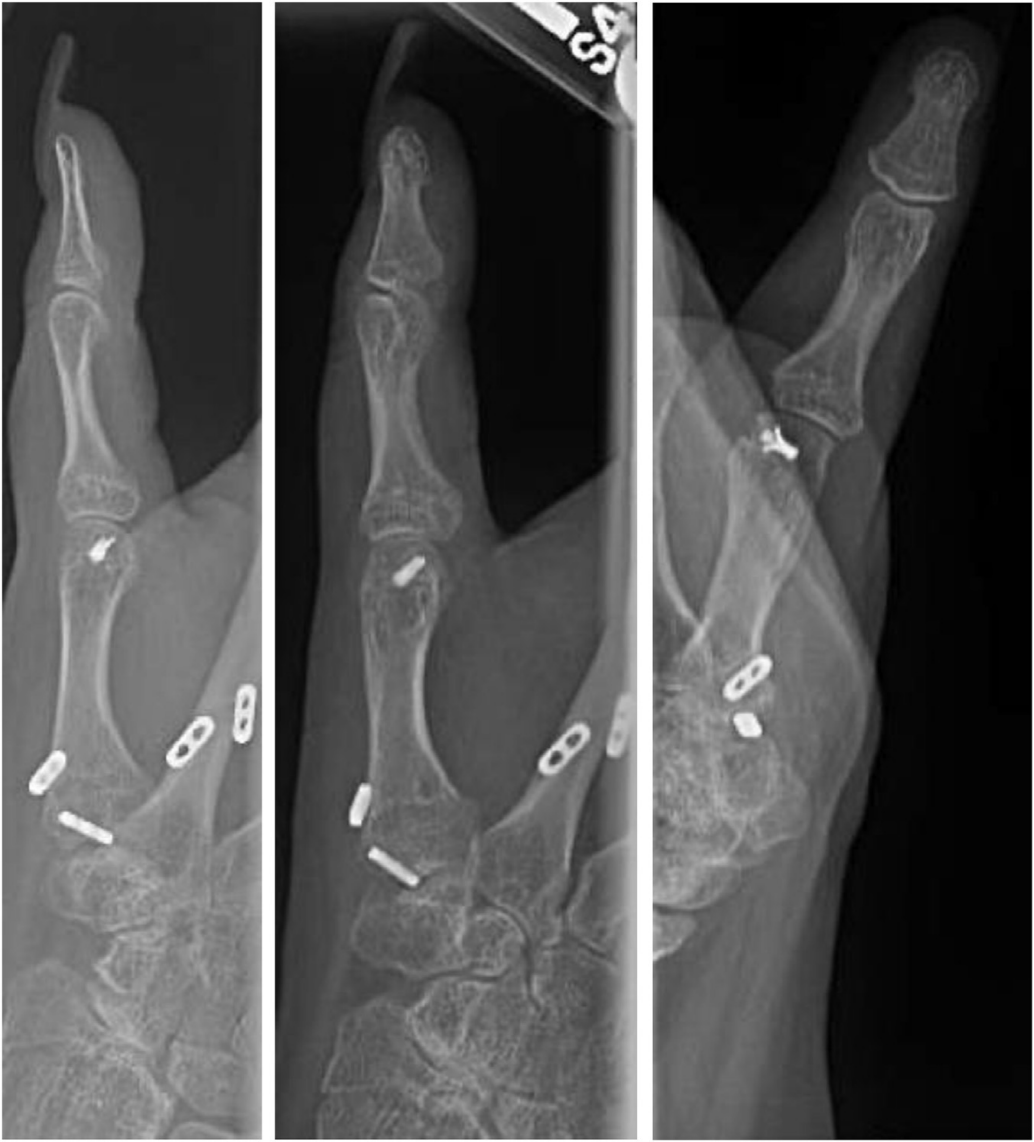
Two-year postoperative posteroanterior, oblique and lateral right thumb radiographs demonstrating stable alignment of the thumb CMC joint with interval reconstruction of the UCL of the MCP joint.

Because of persistent pain and instability at her thumb MCP joint with clinical evidence of UCL insufficiency, she underwent reconstruction of the UCL with a palmaris longus autograft 1 year after surgery. At the final follow-up 2 years after her CMC stabilization (1 year after UCL reconstruction), the patient was free of pain and had returned to all activities. Her thumb MCP joint was stable to varus and valgus stress, and radiographs demonstrated anatomic alignment of both the thumb MCP and CMC joints (Fig. 5).

### Case B

A 36-year-old right hand–dominant female yoga instructor presented with a 2-year history of left base of thumb pain that interfered with her ability to work and complete daily activities. The patient was initially managed with immobilization, anti-inflammatories, and thumb CMC corticosteroid injections at an outside institution. She was evaluated by a rheumatologist, who diagnosed inflammatory arthritis and initiated treatment with prednisone and hydroxychloroquine, which provided an estimated 30% improvement in her symptoms.

**Figure 6:**
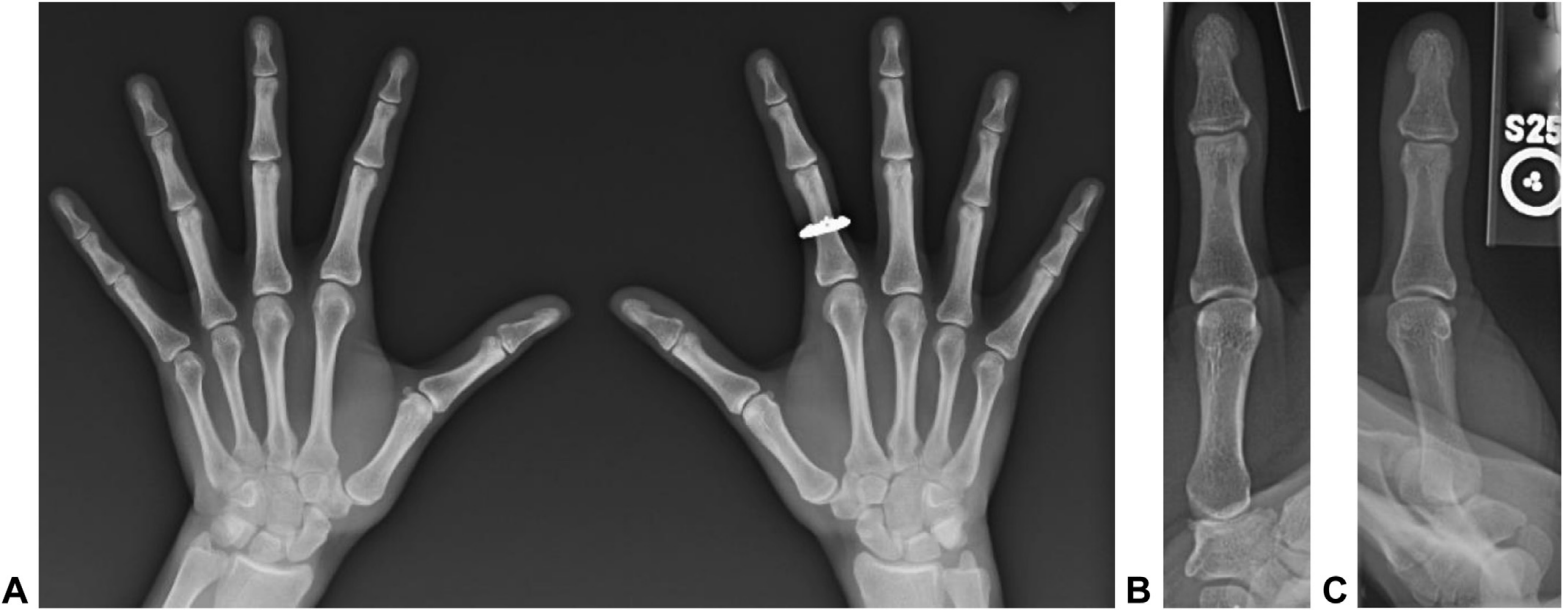
**A** Presenting posteroanterior bilateral hand radiographs left evidence of left thumb CMC radial subluxation with a steep trapezial articular slope. **B** and **C** Dedicated posteroanterior and oblique left thumb radiographs with evidence of mild CMC joint space narrowing and osteophytosis.

**Figure 7:**
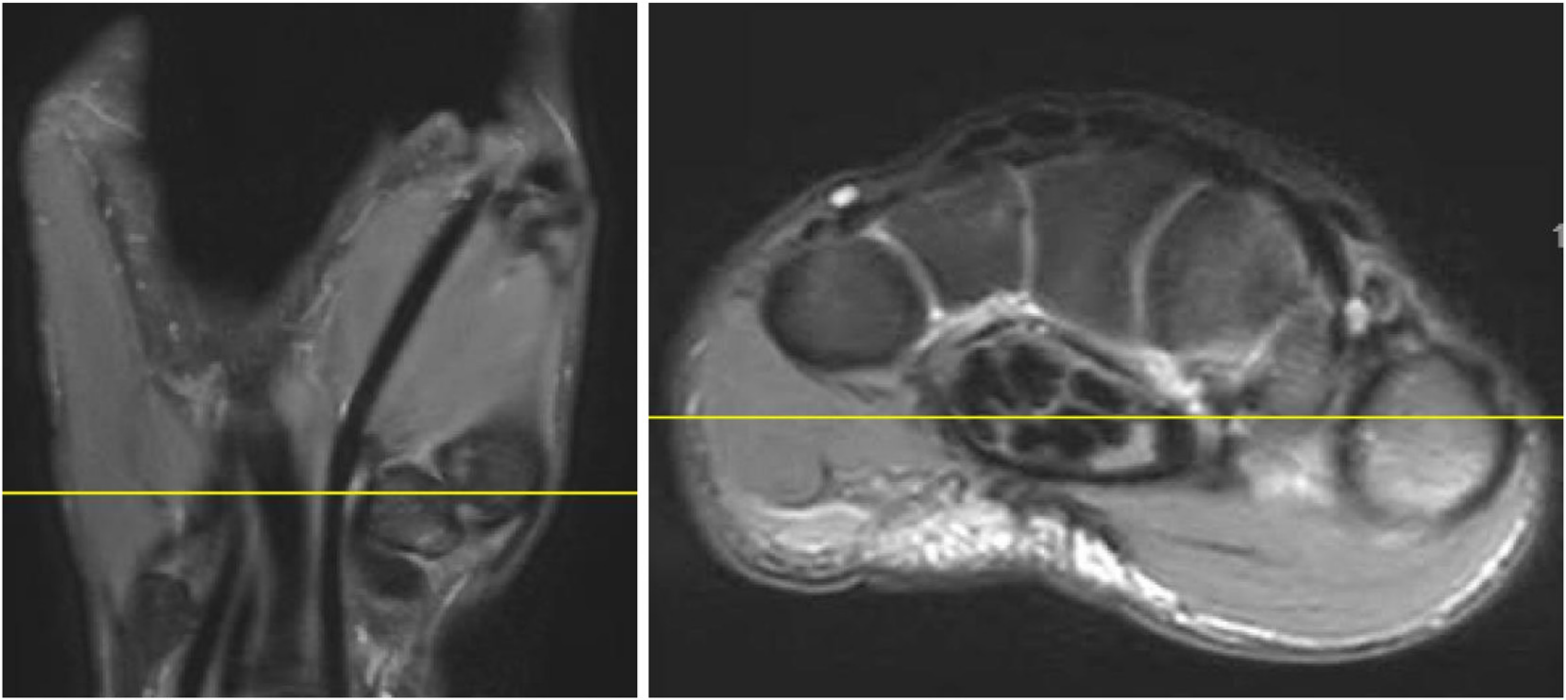
Cross-referenced coronal (left) and axial (right) sections of a proton density sequence of the left hand demonstrating erosive thumb CMC changes with volar osteophytosis, subchondral stress edema, and degeneration of the capsuloligamentous complex.

On our evaluation, she had generalized hyperlaxity with hyperextension of both elbows and knees. She had maximal tenderness to palpation over the thumb CMC joint with evidence of subluxation and a positive grind test. Radiographs demonstrated radial subluxation with mild CMC arthritis, volar osteophytosis, and subchondral stress edema seen on magnetic resonance imaging (Figure 6, Figure 7).

Surgical options with arthrodesis, arthroplasty, and stabilization were discussed with the patient. Given her profession as a yoga instructor, the patient declined arthrodesis as this would prevent her from placing her hand flat. She was deemed a poor candidate for CMC arthroplasty given her young age. Stabilization with Eaton-Littler reconstruction versus a Mini TightRope was discussed, and the patient elected to proceed with the latter treatment option.

#### Surgical technique

Surgical exposure to the thumb CMC joint was similar to that described in the first patient. There was focal cartilage loss along the central metacarpal base and approximately 50% cartilage loss on the trapezium. An Arthrex Mini Tightrope was placed from the first metacarpal base exiting along the ulnar border of the second metacarpal with the thumb held manually suspended approximately 3 mm from the articular surface of the trapezium due to the cartilage loss. With the thumb in position, the Mini TightRope was tensioned, and the thumb was noted to have excellent range of motion without subluxation to manual stress. The patient was immobilized in a thumb spica splint.

#### Postoperative course

**Figure 8:**
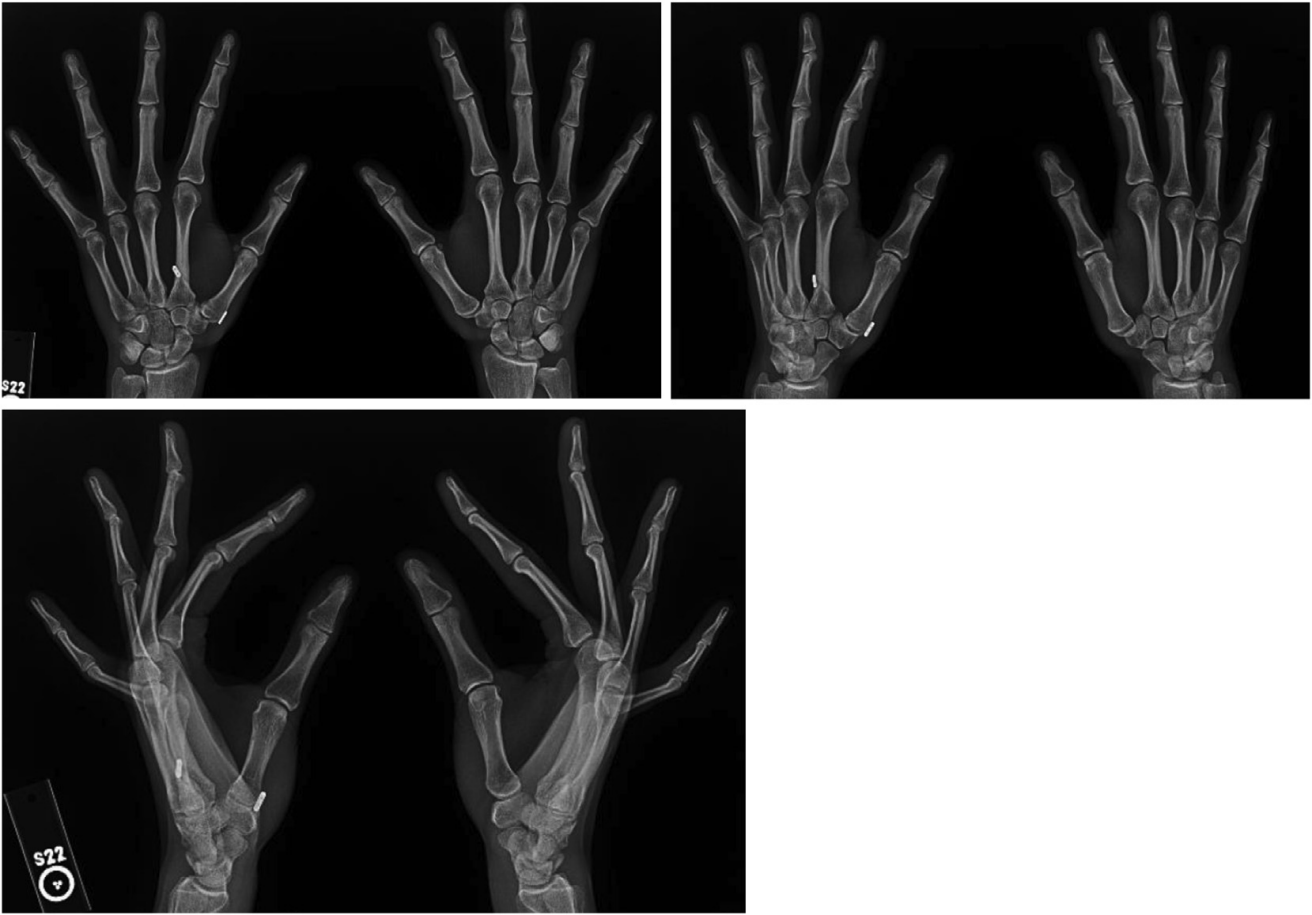
Fifteen-month postoperative posteroanterior, oblique and lateral bilateral hand radiographs with improved left thumb CMC alignment.

On postoperative day 12, the patient was transitioned to a hand-based removable thumb CMC orthosis with instructions to begin gentle thumb opposition exercises. Splint use was weaned 6 weeks after surgery, and strengthening exercises were initiated at 3 months. The patient was allowed to return to teaching yoga 9 months after surgery and had significant improvement in her pain and function. At her latest follow-up 15 months after surgery, she was able to oppose her thumb to the base of the small finger (Kapandji score 10) and had radiographic evidence of anatomic thumb CMC joint alignment (Fig. 8).

## Discussion

Thumb CMC instability may result from several diverse pathologies, including connective tissue disorders, trauma, congenital malformations, and chronic overuse.^3^ Instability of the thumb CMC joint alters joint contact pressure and has been linked to early basilar thumb arthritis.^7^^,^^8^ Regardless of etiology, goals of treatment are directed at creating a stable, pain-free thumb that recreates the native joint contact forces.

A treatment mainstay of thumb CMC instability is the Eaton-Littler stabilization with autologous tendon graft.^4^ This technique has been shown to provide reliable pain relief in 74% to 95% of patients. However, the possibility of a known or suspected connective tissue disorder raises concern for the long-term viability of the reconstruction.^8^ Use of a suture suspension for stabilization of the thumb CMC joint, however, obviates the concern for tendon laxity and degradation. As demonstrated in this report, use of this technique provided reliable pain relief and improvement in radiographic alignment without the need for autologous tendon harvest. Two suture suspension devices were used in case A given the patient’s young age and high functional demands with good results, which aligns with previous reports in the literature.^9^ Further research is needed to determine the optimal indications for one versus two suspensionplasties.

Regardless of technique, timing of intervention is of paramount importance as joint stabilization has decreasing efficacy with advanced stages of arthritis.^1^^,^^8^ As demonstrated with patient B, subluxation of the CMC joint predisposes the patient to early-onset arthritis. Arthrodesis of the thumb CMC joint has been shown to provide reliable pain relief and strength for end-stage arthritis.^10^ However, as demonstrated with patient A, concomitant ligamentous injuries to the thumb MCP joint are not uncommon in this patient population, and arthrodesis of both the thumb MCP and CMC joints drastically decreases function.

Suture suspension as a treatment option for thumb CMC instability with an intact trapezium is a potential alternative to ligamentous reconstruction that avoids donor site morbidity and may have added benefit in patients with underlying ligamentous laxity. Timing of surgical intervention must balance optimization of nonsurgical modalities with the prevention of the development of early arthritic joint changes.
